# Type 1 Autoimmune Pancreatitis Can Transform into Chronic Pancreatitis: A Long-Term Follow-Up Study of 73 Japanese Patients

**DOI:** 10.1155/2013/272595

**Published:** 2013-05-16

**Authors:** Masahiro Maruyama, Norikazu Arakura, Yayoi Ozaki, Takayuki Watanabe, Tetsuya Ito, Suguru Yoneda, Masafumi Maruyama, Takashi Muraki, Hideaki Hamano, Akihiro Matsumoto, Shigeyuki Kawa

**Affiliations:** ^1^Department of Gastroenterology, Shinshu University School of Medicine, 3-1-1 Asahi, Matsumoto 390-8621, Japan; ^2^Endoscopic Examination Center, Shinshu University School of Medicine, 3-1-1 Asahi, Matsumoto 390-8621, Japan; ^3^Center for Health, Safety, and Environmental Management, Shinshu University, 3-1-1 Asahi, Matsumoto 390-8621, Japan

## Abstract

Some patients with autoimmune pancreatitis (AIP) form pancreatic stones suggestive of transformation into chronic pancreatitis (CP). The present study examined the underlying risk factors and mechanism of AIP progression to confirmed CP. We compared the clinical and laboratory parameters of subjects who progressed to confirmed CP with those of the subjucts who did not in a cohort of 73 type 1 AIP patients. A total of 16 (22%) AIP patients progressed to CP. Univariate analysis revealed that relapse was significantly more frequent in the progression group, and multivariate analysis indicated that pancreatic head swelling (OR 12.7, *P* = 0.023) and nonnarrowing of the main pancreatic duct in the pancreatic body (OR 12.6, *P* = 0.001) were significant independent risk factors for progression to CP. Kaplan-Meier testing showed that the progression rate to CP was approximately 10% at 3 years and 30% at 10 years in total AIP patients and 30% at 3 years and 60% at 10 years in subjects with both risk factors. AIP with pancreatic head swelling and a history of relapse may cause pancreatic juice stagnation and nonnarrowing of the main pancreatic duct in the pancreatic body, which can progress to advanced stage chronic pancreatitis.

## 1. Introduction

 Autoimmune 

pancreatitis (AIP) has been recognized as a distinctive type of pancreatitis possibly caused by autoimmune mechanisms [1–3]. Recently, AIP was classified into type 1 and type 2 based on the pathological differences, in which type 1 was designated as lymphoplasmacytic sclerosing pancreatitis (LPSP) and type 2 as idiopathic duct centric chronic pancreatitis (IDCP) or AIP with granulocytic epithelial lesion (GEL) [4–7]. Although the International Consensus Diagnostic Criteria (ICDC) [8] first enabled us to diagnose type 1 and type 2 AIP, AIP in Japan has revealed to be type 1 AIP exclusively. Along with this, all AIP patients in our institution have been diagnosed with type 1 by ICDC, and we have focused on the clinical study for type 1 AIP.Accordingly, in this paper, we dealt with type 1 AIP as AIP.

AIP is characterized by pancreatic enlargement and irregular narrowing of the main pancreatic duct (MPD), both of which resemble the imaging features of pancreatic cancer [9, 10]. Other characteristic features of AIP include high serum IgG4 and IgG4-positive plasma cell infiltration in affected pancreatic tissue, which are used in serological and pathological AIP diagnosis, respectively [11, 12]. As patients with AIP respond favorably to prednisolone (PSL) therapy, the disease was previously believed to be a nonprogressive condition that did not deteriorate into an advanced stage of chronic pancreatitis (CP) or pancreatic stone formation [9]. However, the short-term pancreatic swelling and severe lymphoplasmacytic infiltration seen in acute phase AIP are now believed to manifest as different clinical features in a chronic state; mounting evidence has shown that AIP can progress to an advanced stage, with pancreatic stone formation and atrophy that mimic ordinary CP [13–21].

 Two major mechanisms attempt to explain the pancreatic stone formation observed in AIP: calcification after severe inflammation or tissue necrosis specific to AIP and stasis of pancreatic juice due to irregular narrowing of the pancreatic duct [13]. Concerning the latter, we previously reported that pancreatic stones of any size developed in 53% (37/69) of AIP patients within 3 years primarily due to narrowing of both Wirsung's and Santorini's ducts at the time of diagnosis [22]. 

 The diagnosis of ordinary CP in Japan is based on the revised Japanese clinical diagnostic criteria for chronic pancreatitis [23], in which severe pancreatic stone formation and marked calcification are the main diagnostic criteria. Ordinary CP is also known to be associated with endo- and exocrine dysfunction and severe fibrosis. Some AIP patients appear to progress to confirmed CP with symptoms of severe calcification, but the frequency, pathophysiology, and risk factors of this transformation over a long-term course remain unclear.

 In the present study, we compared the clinical and laboratory parameters of AIP patients with or without progression to confirmed CP to clarify the susceptibility factors and underlying mechanisms for AIP progressing to chronic pancreatitis. 

## 2. Materials and Methods

### 2.1. Study Subjects

Ninety-seven patients with AIP were examined and treated at Shinshu University Hospital between August 1992 and May 2012. Of these, we enrolled 73 patients who had been followed for at least 3 years (median follow-up period: 88 months, range: 36–230 months), which included 56 men and 17 women (median age: 66 years, range: 38–84 years). AIP diagnosis was based on the Asian Diagnostic Criteria for Autoimmune Pancreatitis [24]. In addition, all AIP patients were diagnosed with type 1 AIP by ICDC [8].

### 2.2. Diagnostic Criteria for Chronic Pancreatitis

We investigated the progression of AIP to confirmed definite or probable CP in terms of the revised Japanese clinical diagnostic criteria for chronic pancreatitis [23] that are listed in Table 1. This study did not evaluate MRCP or US (EUS) findings, so the probable CP criteria imaging findings using these modalities were excluded, namely, “(a) irregular dilatation of the MPD and irregular dilatation of pancreatic duct branches of variable intensity with scattered distribution throughout the entire pancreas on MRCP” and “(d) intra-pancreatic coarse hyperreflectivities suggestive of stones or protein plugs or irregular dilatation of pancreatic ducts plus pancreatic deformity with irregular contour on US (EUS).”

### 2.3. Clinical Features and Laboratory Tests

We reviewed the medical records of our cohort for comparisons of observation period, age at diagnosis, gender, alcohol consumption (ethanol > 25 g/day), PSL treatment, PSL maintenance therapy, and relapse between AIP patients who did or did not progress to CP. We also compared serum values of AIP activity markers at diagnosis, including IgG, IgG4, C3, C4, soluble interleukin 2 receptor (sIL2-R), and circulating immune complex (CIC). 

### 2.4. Evaluation of Pancreatic Stone Formation

The presence of pancreatic stones was assessed by CT images. CT scanning was performed using different protocols during the course of this study; CT testing was changed to multidetector computed tomography (MDCT) at our institute in 2003, which resulted in clearer images.

### 2.5. Evaluation of Pancreatic Swelling

Swelling of the pancreas in CT images was assessed by 3 pancreatology experts. Pancreatic swelling was considered to be present using the Haaga criteria [25] or by a marked decrease in size after PSL therapy. Swelling was classified as level 1 (diffuse swelling) or level 2 (focal-segmental swelling) as defined by the International Consensus Diagnostic Criteria for Autoimmune Pancreatitis (ICDC) [8].

### 2.6. Evaluation of Pancreatic Duct Images

Pancreatic duct images from endoscopic retrograde pancreatocholangiography (ERCP) were assessed by 3 endoscopic experts. Normal MPD diameter was defined as approximately 2-3 mm. MPD narrowing was defined as being “unlike obstruction or stenosis, the narrowing extends to a certain degree and the duct diameter is smaller than normal, with some irregularities” [26]. Dilatation of the MPD was defined as a diameter of 4 mm or more. Pancreatic duct narrowing was classified as level 1 (long (>1/3 the length of the MPD) or multiple narrowing) or level 2 (focal (<1/3 the length of the MPD) narrowing), as outlined by the ICDC [8].

### 2.7. Statistical Analysis

Fisher's exact and Pearson's chi-square tests were adopted to test for differences between the subgroups of patients. The Mann-Whitney *U* test was employed to compare continuous data. Multivariate analyses were performed using a logistic regression model. Variables associated with a *P* value of <0.2 in univariate analyses were included in a stepwise logistic regression analysis to identify independent risk factors associated with the progression to CP. The Kaplan-Meier method was used for analysis of AIP transformation into CP. All tests were performed using the IBM SPSS Statistics Desktop for Japan ver. 19.0 (IBM Japan Inc, Tokyo, Japan). *P* values of less than 0.05 were considered to be statistically significant.

### 2.8. Ethics

This study was approved by the ethics committee of Shinshu University (approval number 1973).

## 3. Results

### 3.1. Progression to Chronic Pancreatitis

**Figure 1:**
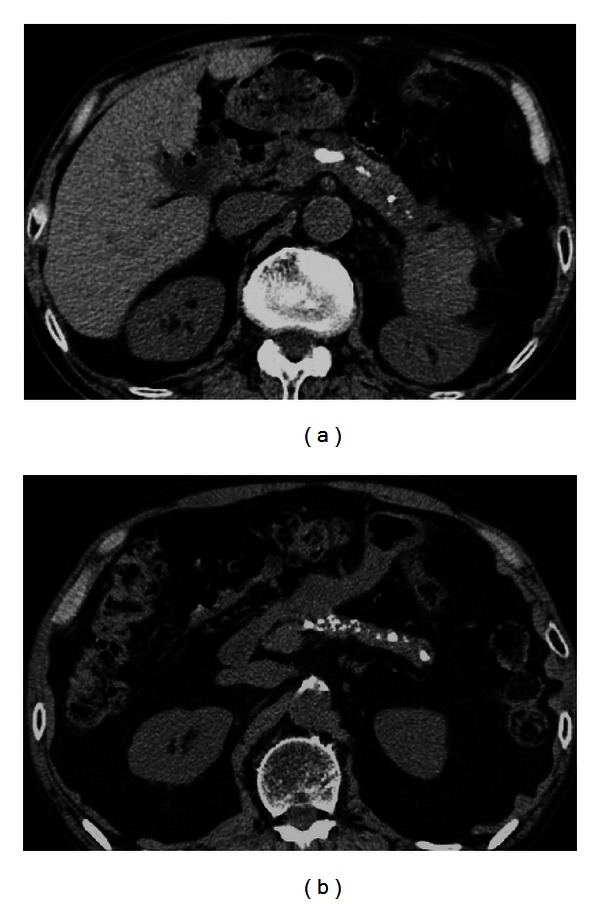
CT of AIP showing definite imaging findings. (a) Stones in pancreatic ducts. (b) Multiple or numerous calcifications distributed throughout the entire pancreas.

 During the study period, 16 (22%) patients with AIP progressed to confirmed CP, which included 15 patients with definite CP and 1 patient with probable CP (Table 1). Among the 15 definite CP patients, imaging findings were stones in pancreatic ducts in 9 patients (Figure 1(a)), multiple or numerous calcifications distributed throughout the entire pancreas in 13 patients (Figure 1(b)), irregular dilatation of the MPD and irregular dilatation of pancreatic duct branches of variable intensity with scattered distribution throughout the entire pancreas on ERCP in 2 patients, and irregular dilatation of the MPD and branches proximal to complete or incomplete obstruction of the MPD (with pancreatic stones or protein plugs) on ERCP in 2 patients. The imaging finding of irregular dilation of the MPD alone was found in the single case of probable CP (Table 1).

### 3.2. Correlation between Chronic Pancreatitis Diagnosis and Clinical and Laboratory Features Associated with Autoimmune Pancreatitis Activity

We next searched for risk factors attributed to progression to confirmed CP by comparing clinical and laboratory parameters between AIP patients who progressed to CP (*n* = 16) with those who did not (*n* = 57). Univariate analysis revealed no significant differences in observation period, age at diagnosis, gender, alcohol consumption, PSL therapy, or PSL maintenance therapy between the two groups. However, relapse (*P* = 0.030) was significantly more frequent in the progression group. We found no significant differences in serum values of the AIP activity markers IgG, IgG4, C3, C4, sIL2-R, or CIC between the two groups (Table 2).

### 3.3. Correlation between Chronic Pancreatitis Diagnosis and Pancreatic Swelling

 We examined whether progression to confirmed CP was associated with the extent (level 1 versus level 2) or location of pancreatic swelling. Univariate analysis showed no significant differences in the extent of pancreatic swelling between the two groups. Pancreatic head swelling (*P* = 0.096) was more frequently seen in the progression group, albeit not significantly (Table 2). 

### 3.4. Correlation between Chronic Pancreatitis Diagnosis and Pancreatic Duct Images

 We next examined whether progression to confirmed CP was associated with the extent (level 1 versus level 2) or location of MPD narrowing or with MPD dilatation at one pancreatic area or more. Univariate analysis revealed no significant differences in the extent of MPD narrowing between two groups. However, MPD narrowing in the pancreatic body was significantly less frequent (*P* = 0.001), and MPD dilatation at one pancreatic area or more was significant more frequently (*P* = 0.001), in the progression group (Table 2). Thirteen AIP patients with nonnarrowing of the main pancreatic duct (MPD nonnarrowing) in the pancreatic body are included: 8 patients with dilated duct diameter and 5 with normal one. All of the 8 patients with dilated duct diameter had pancreatic head swelling, in which 7 had diffuse swelling. Five patients had normal duct diameter: 3 patients with diffuse swelling, 1 with only head swelling, and 1 with only tail swelling. None of the 13 patients with MPD nonnarrowing in the pancreatic body had any pancreatic atrophy.

### 3.5. Multiple Logistic Regression Analysis of Factors Associated with Progression to Chronic Pancreatitis

**Figure 2:**
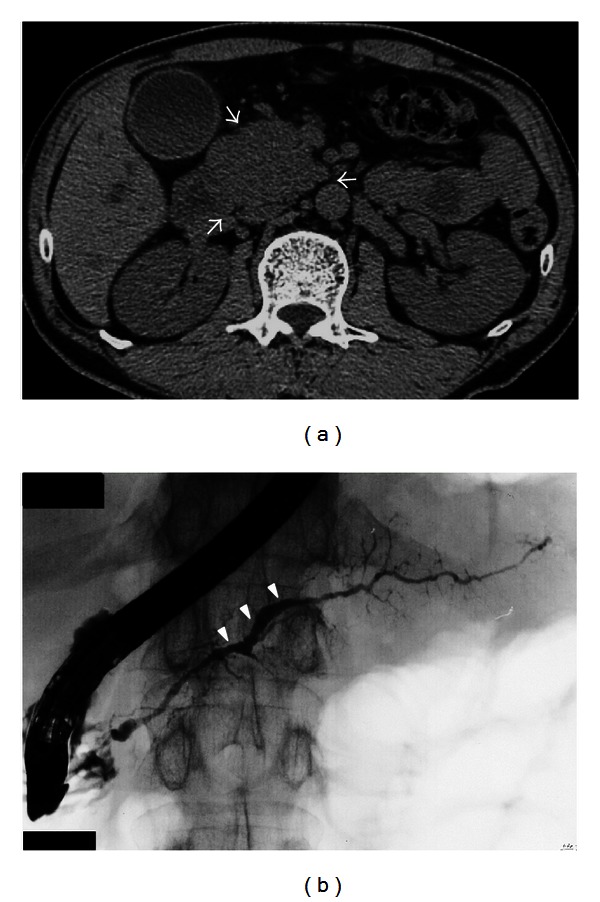
CT and ERCP findings of AIP showing independent risk factors for progression to confirmed chronic pancreatitis at diagnosis. (a) Pancreatic head swelling (arrows). (b) MPD nonnarrowing in the pancreatic body (arrowheads).

 Multiple logistic regression analysis was performed for relapse, pancreatic head swelling, MPD nonnarrowing in the pancreatic body, and MPD dilatation at one pancreatic area or more; all of which had *P* values of less than 0.2 in univariate studies. We identified that pancreatic head swelling was a significant independent risk factor for progression to confirmed CP (Odds ratio: 12.7, 95% confidence interval: 1.4–114.5, *P* = 0.023) (Figure 2(a)), as was MPD nonnarrowing in the pancreatic body (odds ratio: 12.6, 95% confidence interval: 3.003–52.6, *P* = 0.001) (Figure 2(b)) (Table 3).

### 3.6. Progression of Autoimmune Pancreatitis to Chronic Pancreatitis

**Figure 3:**
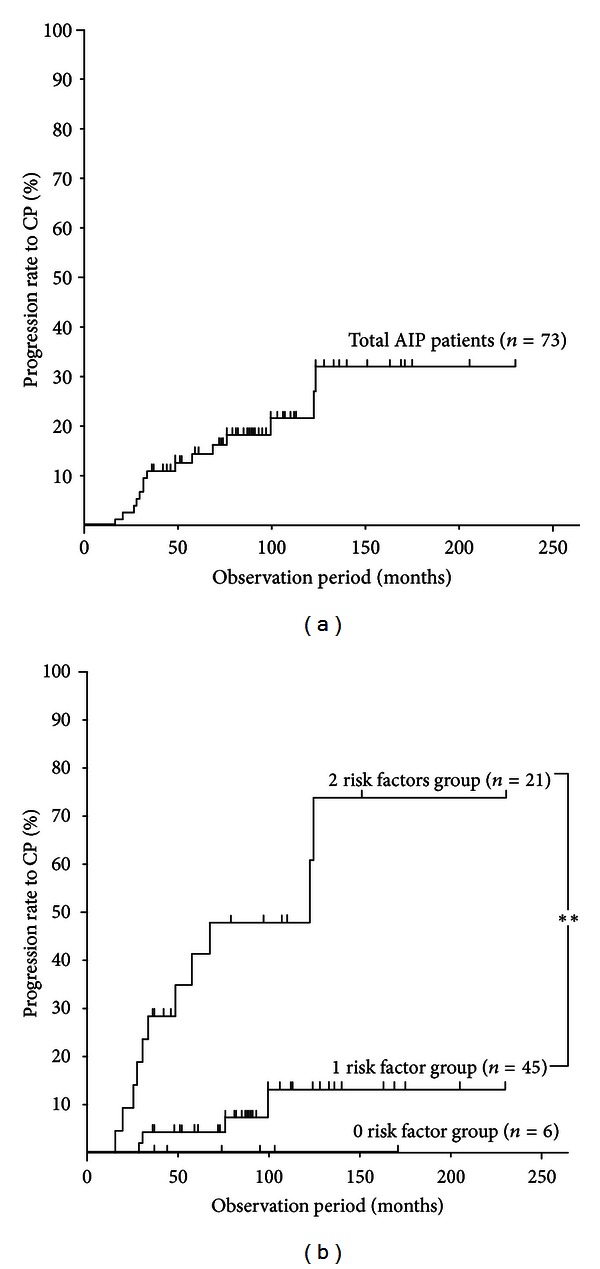
(a) Kaplan-Meier analysis of the progression rate to confirmed chronic pancreatitis in 73 patients with AIP. (b) Kaplan-Meier analysis of the progression rate to confirmed chronic pancreatitis in AIP based on the risk factors of pancreatic head swelling and MPD nonnarrowing in the pancreatic body. Comparison of the zero risk factor (*n* = 6), 1 risk factor (*n* = 45), and 2 risk factors (*n* = 21) groups. **P < 0.001 (log-rank test).

The median time from AIP diagnosis to confirmed CP was 33 months (range: 16–124 months). Kaplan-Meier testing revealed that the transformation rate into CP was 10% at 36 months, 20% at 100 months, and 30% at 124 months. No new cases of CP were noted from 124 months to the end of the observation period (Figure 3(a)).

Stratification analysis for AIP transformation into confirmed CP was performed using the two risk factors identified by multiple regression analysis of pancreatic head swelling and MPD nonnarrowing in the pancreatic body. Specifically, Kaplan-Meier evaluation was performed on 3 groups: the zero risk factor group (6 patients), the 1 risk factor group (45 patients), and the two risk factors group (21 patients). No AIP patients progressed to confirmed CP in the zero risk factor group, whereas the 2 risk factors group showed a significantly higher transformation rate compared with that of the 1 risk factor group (*P* < 0.001, log-rank test) of 30% at 3 years and 60% at 10 years (Figure 3(b)). 

## 4. Discussion

 Twenty-two percent of the AIP patients in our long-term follow-up cohort progressed to CP that met the Japanese diagnostic criteria for ordinary chronic pancreatitis. To our knowledge, this is the first study to demonstrate such a transformation into advanced stage of CP with severe calcification. Previous reports showed that AIP developed morphological changes of pancreatic stone formation and atrophy that were closely associated with endo- and exocrine function insufficiency over a long-term course, suggesting that AIP had the potential to progress to a chronic state resembling ordinary CP [27, 28]. A French study disclosed that more than one-third of AIP patients developed pancreatic imaging abnormalities of atrophy, calcification, and/or duct irregularities and functional insufficiency within 3 years of diagnosis. Specifically, endo- and exocrine function insufficiency occurred in 57% and 36% of type 1 AIP patients, respectively, during a median follow-up period of 41 months. Corticosteroid treatment could not prevent the pancreatic insufficiencies in the group [21]. We once found that 7% of patients with apparently typical CP also had elevated serum IgG4 concentration, which may have in fact represented chronic stage AIP [29]. However, other studies showed low rate of pancreatic stone formation during long-term followup of AIP compared with ours [15, 30], and further studies are needed to disclose these discrepancy.

 We earlier reported that the primary risk factors for pancreatic stone formation during AIP followup were narrowing of both Wirsung's and Santorini's ducts [22]. In this study, we confirmed that AIP patients could form severe pancreatic stones in the main pancreatic duct or throughout the entire pancreas and evaluated the risk factors that contributed to AIP progression to definite/probable CP. By comparing progression with nonprogression patients, univariate analysis disclosed that relapse and MPD dilatation were significantly more frequent in the progression group. Pancreatic head swelling was also more frequently seen in this group, albeit not significantly. MPD narrowing in the pancreatic body was significantly less frequent in the progression group. Multivariate analysis confirmed that pancreatic head swelling and MPD nonnarrowing in the pancreatic body were significant independent risk factors in the progression group, with the latter factor also implying that normal or dilatated MPD diameters in this region may be significant independent risk factors for progression to CP during AIP followup. We believe that the MPD nonnarrowing in the pancreatic body reflects increased intrapancreatic duct pressure due to downstream pancreatic duct narrowing in the head region. In fact, almost all patients with MPD nonnarrowing in the pancreatic body had pancreatic head swelling, in which dilated MPD diameters in this region might represent high intrapancreatic duct pressure due to severe duct stricture of head region and normal diameter might represent mild intrapancreatic duct pressure or mild duct compression by pancreatic body swelling. Furthermore, none of all patients with MPD nonnarrowing in the pancreatic body had any pancreatic atrophy; therefore it was less likely that nonnarrowing of the main pancreatic duct in the body region represented burnt-out phase of AIP at diagnosis. In this study, narrowing of both Wirsung's and Santorini's ducts wasnot a significant independent risk factor for CP, though these had been confirmed to be independent risk factors for pancreatic stone formation [22]. The reason for this discrepancy may be due to that narrowing of both Wirsung's and Santorini's ducts may be in part related to the small pancreatic calculi which cannot fulfill the diagnostic criteria of confirmed CP and were classified into the nonprogression group to CP.

Univariate analysis disclosed that AIP-specific activity markers, such as IgG, IgG4, C3, C4, sIL2-R, and CIC, were not significantly different between the progression and nonprogression groups, indicating that AIP activity itself had no measurable contribution to progression to confirmed CP. There were also no significant differences in corticosteroid or maintenance treatments. Thus, it appears that once pancreatic juice stasis due to pancreatic duct narrowing is established, AIP develops into severe pancreatic calcification regardless of prior or ongoing treatment. AIP in general responds favorably to corticosteroid therapy, which results in amelioration of pancreatic swelling and MPD narrowing; however, our previous study revealed that pancreatic swelling and MPD narrowing showed tendency to persist in the stone-forming group after therapy compared with the nonstone-forming group [22].

Though the present study showed that alcohol consumption of ethanol >25 g/day was not the risk factor for progression to CP; Hirano et al. reported that high alcohol consumption of ethanol >50 g/day increased the risk of pancreatic stone development and atrophy, indicating that changes of pancreatic juice character due to high alcohol consumption may in part contribute to stone formation in AIP [31]. We were not able to identify correct reasons for discrepancy between Hirano's results and ours. Higher volume consumption of ethanol (ethanol > 50 g/day) found in Hirano's study might result in more lithogenic nature of pancreatic juice. Further study is needed using the same criteria of alcohol consumption of ethanol >50 g/day.

 The overall transformation rate into confirmed CP was 10% at 36 months, 20% at 100 months, and 30% at 124 months. Transformation into confirmed CP was not seen after 124 months, suggesting that the window for disease development is within 10 years of followup. We also performed Kaplan-Meier testing on AIP transformation based on the 2 independent risk factors of pancreatic head swelling and MPD nonnarrowing in the pancreatic body. AIP patients without these risk factors were far less likely to progress to confirmed CP, as evidenced by no transformation in the zero risk factor group. In contrast, the 2 risk factors group showed a significantly higher frequency of transformation compared with the 1 risk factor group of 30% at 3 years and 60% at 10 years. At present, standard initiation criteria for steroid therapy in Japan may represent obstructive jaundice and any symptoms such as abdominal pain, and many patients had maintenance therapy of over 3 years to prevent recurrence based on the Japanese consensus guideline for AIP, though variety of therapeutic regimen have been employed in each institute. It is necessary to construct effective regimen to protect the progression to chronic pancreatitis. Early intensive care and sufficient maintenance therapy for AIP patients with 2 risk factors may result in the prevention for the progression into chronic pancreatitis.

**Figure 4:**
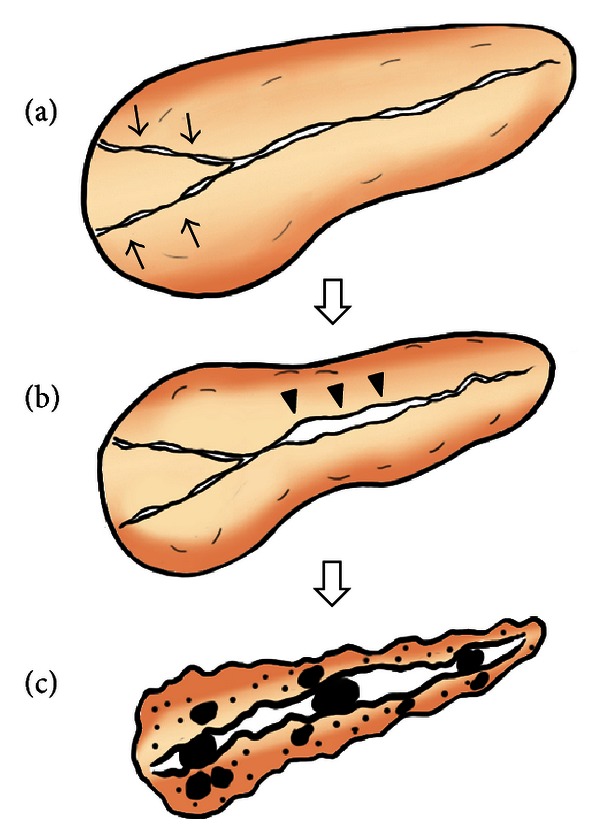
Sequential progression mechanism of AIP to confirmed chronic pancreatitis. (a) Narrowing of both Wirsung's and Santorini's ducts (arrows) by pancreatic head swelling causes pancreatic juice stasis in the upstream pancreatic duct. (b) Pancreatic juice stasis results in increased intrapancreatic duct pressure, that is, resistance to typical AIP-specific MPD narrowing in the pancreatic body region, leading to MPD nonnarrowing in this region (arrowheads). (c) These events finally result in severe calcification.

Based on our cumulative findings, we propose the following sequential progression mechanism of AIP to confirmed CP: pancreatic head swelling and narrowing of both Wirsung's and Santorini's ducts cause pancreatic juice stasis in the upstream pancreatic duct, which results in increased intrapancreatic duct pressure, that is, resistance to typical AIP-specific MPD narrowing in the pancreatic body, causing MPD nonnarrowing in this region. These events finally result in severe calcification of the entire pancreas (Figure 4). In this study, we found only one patient with focal-type AIP which involved tail portion without head involvement among 16 patients who progressed to chronic pancreatitis. Because this patient was alcoholic (daily ethanol consumption > 80~100 g), major cause for progression to chronic pancreatitis may be alcohol abuse.

Limitations of the present study are as follows: the study design was retrospective cohort one, we applied Japanese diagnostic criteria for CP with particular reference to image findings, and AIP patients were biased as type 1. Because we focused on the study for image findings, detailed analysis for exocrine or endocrine dysfunction and pathological findings is needed in future study.

 In conclusion, this study established that AIP patients having pancreatic head swelling and/or MPD nonnarrowing in the pancreatic body may progress to an advanced stage of CP due to pancreatic juice stagnation.

## Figures and Tables

**Table 1:** Breakdown of the diagnostic imaging findings for chronic pancreatitis as determined by the revised Japanese clinical diagnostic criteria for chronic pancreatitis.

	Number
Findings of definite chronic pancreatitis (*n* = 15)	
(a) Stones in pancreatic ducts	9
(b) Multiple or numerous calcifications distributed in the entire pancreas	13
(c) Irregular dilatation of the MPD and irregular dilatation of pancreatic duct branches of variable intensity with scattered distribution throughout the entire pancreas on ERCP	2
(d) Irregular dilatation of the MPD and branches proximal to complete or incomplete obstruction of the MPD (with pancreatic stones or protein plugs) on ERCP	2
Findings of probable chronic pancreatitis (*n* = 1)	
(b) Irregular dilatation of pancreatic duct branches of variable intensity with scattered distribution throughout the entire pancreas, irregular dilatation of the MPD alone, or protein plugs on ERCP	1
(c) Irregular dilatation of the MPD throughout the entire pancreas plus pancreatic deformity with irregular contour on CT	0

This study did not evaluate MRCP or US (EUS) findings, so the probable chronic pancreatitis findings of (a) and (d), which are judged by these modalities, were excluded.

**Table 2:** Clinical features, laboratory tests, and pancreatic morphology at diagnosis.

	Progression to CP (*n* = 16)	Nonprogression to CP (*n* = 57)	*P* value
Clinical features	Median (range)		
Observation period^†^	102 (37–165)	87 (36–230)	0.522
Age	66.5 (48–75)	65 (38–84)	0.989
Gender (M/F)	13/3	43/14	0.748
Alcohol (+/−)	6/10	29/28	0.405
PSL (+/−)	13/3	50/7	0.681
PSL maintenance therapy (+/−)	10/6	41/16	0.542
Relapse (+/−)	8/8	12/45	0.030*
Laboratory tests			
IgG	2140 (1166–3861)	2227 (892–7236)	0.509
IgG4	421 (146–1845)	663 (4–2970)	0.267
C3	100 (52–122)	98 (29–218)	0.551
C4	21.8 (12.4–37.7)	21.1 (1.1–47.3)	0.495
sIL2-R	726 (132–1845)	892 (257–4695)	0.053
CIC	5 (1.9–13.9)	5.7 (1.4–40)	0.219
Pancreatic morphology at diagnosis			
Pancreatic swelling			
Head (+/−)	15/1	41/16	0.096
Body (+/−)	12/4	36/21	0.553
Tail (+/−)	10/6	37/20	1.000
Level 1/Level 2^Φ^	8/8	30/27	1.000
Ductal narrowing in MPD			
Head (+/−)	13/3	44/13	1.000
Wirsung and Santorini (+/−)	11/5	34/23	0.573
Body (+/−)	3/13	37/20	0.001*
Tail (+/−)	12/4	42/15	1.000
Level 1 / Level 2^Ψ^	6/10	17/40	0.558
Ductal dilatation in MPD (+/−)	9/7	7/50	0.001*

^†^Period from AIP diagnosis to the most recent observation (months).

^Φ^Swelling was classified as level 1 (diffuse swelling) or level 2 (focal/segmental swelling) as defined by the International Consensus Diagnostic Criteria for Autoimmune Pancreatitis.

^Ψ^Pancreatic duct narrowing was classified as level 1 (long (segmental/diffuse) or multiple strictures) or level 2 (focal narrowing) as defined by the International Consensus Diagnostic Criteria for Autoimmune Pancreatitis.

*P < 0.05.

CP: chronic pancreatitis; PSL: prednisolone; sIL2-R: soluble interleukin 2 receptor; CIC: circulating immune complex; and MPD: main pancreatic duct.

**Table 3:** Multiple regression analysis of factors associated with progression to chronic pancreatitis.

Factor	Odds ratio (95% Confidence interval)	*P* value
Pancreatic head swelling	12.7 (1.40–114.5)	0.023*
MPD nonnarrowing in the pancreatic body	12.6 (3.00–52.6)	0.001*

CP: chronic pancreatitis and MPD: main pancreatic duct.

*P < 0.05.
